# Causal association between blood metabolites and risk of hypertension: a Mendelian randomization study

**DOI:** 10.3389/fcvm.2024.1373480

**Published:** 2024-06-07

**Authors:** Tao Cheng, Zhangjun Yun, Shaowei Fan, Huan Wang, Wenjing Xue, Xuesong Zhang, Bochao Jia, Yuanhui Hu

**Affiliations:** ^1^ Department of Cardiological Medicine, China Academy of Chinese Medical Sciences Guang’anmen Hospital, Beijing, China; ^2^ Clinical Medical College, Beijing University of Chinese Medicine, Beijing, China

**Keywords:** metabolites, essential hypertension, mendelian randomization, causality, N-acetylornithine

## Abstract

**Background:**

Previous studies have indicated a strong link between blood metabolites and hypertension, however the causality of metabolites and hypertension is unknown.

**Methods:**

Two-sample Mendelian randomization (MR) analysis was performed to assess the causal relationship between 486 blood metabolites and essential hypertension (EHT). Blood metabolite GWAS data was utilized as the exposure, with EHT GWAS data as the outcome. To further verify the results, another different source of EHT GWAS data was repeatedly analyzed. The major MR analytic approach used to determine causality was inverse variance weighted (IVW), with MR-Egger, Weighted Median, and MR-PRESSO models serving as supplements. We used the Cochran Q test to examine heterogeneity. Horizontal pleiotropy was examined using MR-Egger intercept and MR-PRESSO global test. The MR Steiger test confirmed the causal relationship between blood metabolites and EHT.

**Results:**

In this study, nine blood metabolites associated with EHT were preliminarily identified by MR analysis, including four known metabolites (N-acetylornithine, X-12510–2-aminooctanoic acid, creatine, hexadecanedioate) and five unknown metabolites. Then another source of EHT GWAS data was repeatedly analyzed for further verification, and two overlapped metabolites (N-acetylornithine, X-12510-2-aminooctanoic acid) were found. There was a negative correlation between N-acetylornithine and EHT (OR = 0.987, 95% CI = 0.980–0.993, *P* = 1.01 × 10^−4^), Cochran's Q test suggested there was no heterogeneity (*Q* = 31.7586, *P* = 0.1331), MR-Egger intercept and MR-PRESSO global test suggested there was no horizontal pleiotropy (*P* > 0.05), Leave-one-out analysis indicated that no single single-nucleotide polymorphism (SNP) had a significant effect on the results, and MR Steiger test confirmed that the direction of causality was correct (*P* < 0.001). There was a negative correlation between X-12510-2-aminooctanoic acid and EHT (OR = 0.982, 95% CI = 0.972–0.993, *P* = 0.0017), and there was no evidence of heterogeneity or pleiotropy in multiple sensitivity analyses.

**Conclusion:**

The study discovered some blood metabolites causally linked to EHT, which might lead to new understandings of the pathophysiology of hypertension.

## 1 Introduction

Hypertension is a cardiovascular disease characterized by the increase of systemic arterial blood pressure (systolic and/or diastolic), which is one of the major global health problems and affects a large number of people every year (1). It has been reported that the prevalence of hypertension is expected to increase to 29.2% by 2025 (2). Hypertension is a major risk factor for cardiovascular complications, cerebrovascular disease, kidney disease and death (3, 4), which brings a significant burden to individuals, families and society. The pathogenesis of essential hypertension (EHT) is very complex. Although there have been many related studies and findings, the mechanisms remain largely unclear.

Metabolites, including sugars, lipids, peptides, amino acids and other biological small biomolecular compounds, are the material foundation of system life and biochemical phenotype, and can provide comprehensive information on the physiological and pathological status and metabolic pathways of the body. Metabolomics is a relatively new research method, which systematically studies the unique chemical map of small molecular compounds related to various metabolic processes in cells, organs, tissues and organisms (5, 6), and offers direct information for studying the pathogenesis of diseases (7). It has been widely used in various scientific fields and is expected to discover new biomarkers related to disease diagnosis and prognosis (8).

Previous studies have explored the correlation between metabolites and hypertension, and found that some metabolites were crucial in the occurrence and development of hypertension. He et al. found 24 metabolites closely related to blood pressure, including three amino acid and nucleotide metabolites, seven cofactors and vitamins or heterotrophic metabolites, ten lipid metabolites and four still unnamed metabolites (9). Bai et al. found that Gly, Orn, C10, Orn/Cit, Phe/Tyr and C5-OH/C8 may be used to distinguish between EHT patients and healthy individuals (10). Lin et al. found that ceramide, triacylglycerol, total glycerolipids, oleic acid and cholesterylester were associated with diastolic blood pressure (11). However, most of these studies are observational, making it difficult to determine the causal association between metabolites and hypertension due to confounding factors and bias.

The fundamental tenet of Mendelian randomization (MR) is to establish a random grouping process similar to Randomized clinical trials (RCT) in the population according to the principle of natural random allocation in the process of meiosis (a way of cell division) in genetics (12), which can be used as an alternative research strategy of RCT, and is capable of overcoming observational research’ inherent bias and unidentified confounding variables (13, 14). In the MR analysis, single nucleotide polymorphisms (SNPs) were used as genetic instrumental variables (IVs), so the MR study can effectively evaluate if there may be a potential causal link between the exposure and the outcome (15).

This study assessed the causal association between blood metabolites and EHT using the two-sample MR method, in order to have a deeper knowledge of the pathophysiology of EHT.

## 2 Methods and materials

### 2.1 Study design

**Figure 1:**
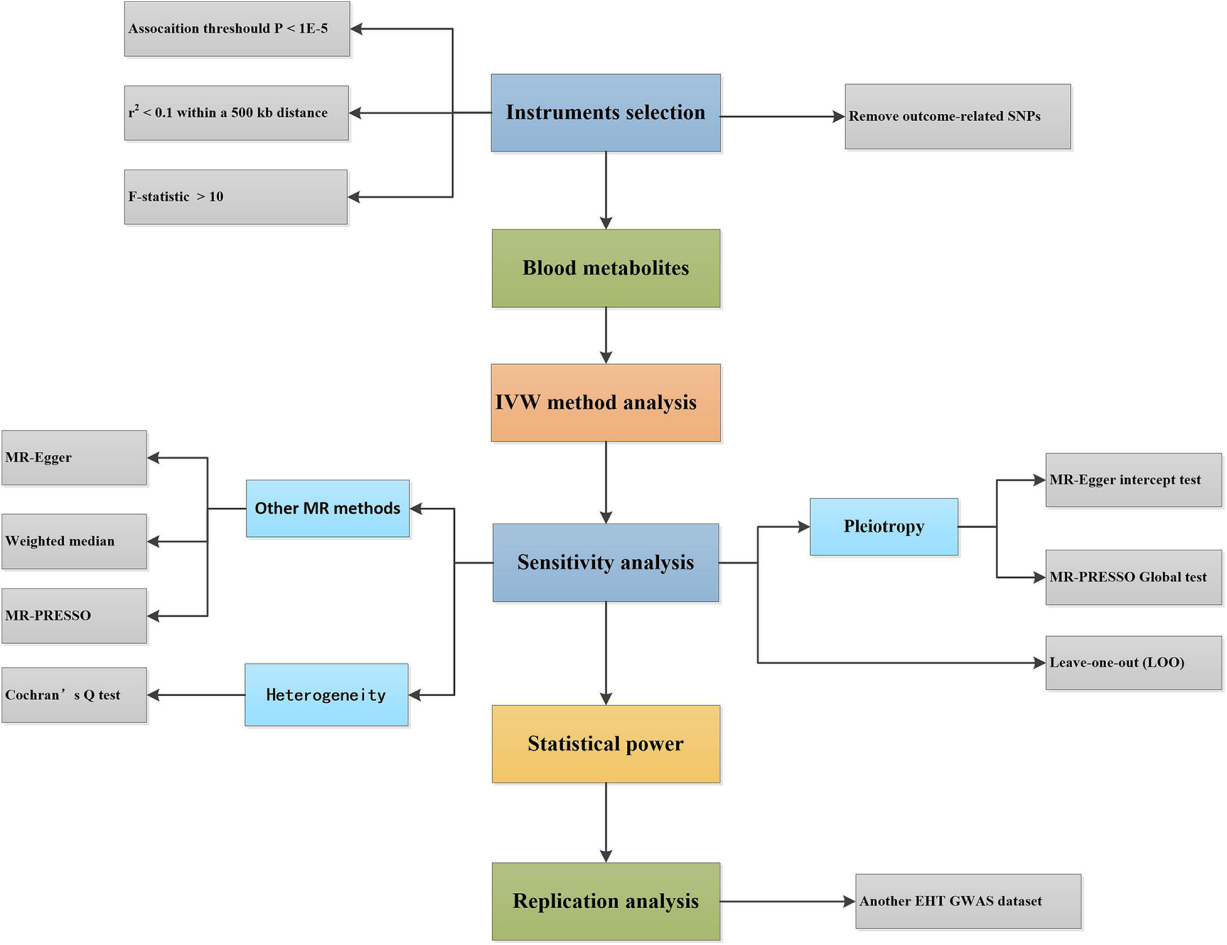
Design flowchart of the Mendelian randomization study. EHT, essential hypertension; IVW, inverse-variance weighted; MR, Mendelian randomization; SNPs, single nucleotide polymorphisms; GWAS, genome-wide association study.

This study used genome-wide association study (GWAS) statistical data and two-sample MR analysis to determine the causal association between 486 blood metabolites and EHT. The flowchart of the study design is shown in Figure 1.

In principle, IVs must meet the following assumptions: (1) IVs are directly connected to the exposure; (2) IVs are not associated with any potential confounding variables; (3) IVs are solely related to the outcome via the exposure (not through other factors) (16).

### 2.2 Source of GWAS data for exposure and outcome

In the study, the GWAS data for exposure and outcome based on European ancestry were all sourced from publicly available online data and have been ethically approved in previous studies (see Table 1).

**Table 1:** Summary of exposure and outcome GWAS data.

Phenotype	Ancestry	Category	Sample size (case/control)	Consortium/Author	Year
Exposure					
Human blood metabolites	European	Continuous	7,824	Shin et al	2014
Outcome					
Essential hypertension	European	Binary	324,894 (81,138/243,756)	FinnGen	2022
Essential hypertension	European	Binary	463,010 (54,358/408,652)	MRC-IEU	2018

#### 2.2.1 GWAS data for exposure

The human blood metabolite GWAS data came from a research that was published (17). Shin et al. conducted a comprehensive and systematic genome-wide association estimation study on blood metabolites in 7,824 adults from Europe (1,768 Germans and 6,056 British). In this study, 486 metabolites were analyzed by the Metabolon platform, including 309 identified metabolites and 177 unidentified metabolites. These identified metabolites were divided into eight types: carbohydrates, lipids, amino acids, polypeptides, nucleotides, cofactors and vitamins, energy products and xenobiotics. About 2.1 million SNPs were retained in the GWAS summary statistics. The data in this study is publicly available on the Metabolomics GWAS server (http://metabolomics.helmholtz-muenchen.de/gwas/).

#### 2.2.2 GWAS data for outcome

FinnGen is a large-scale cooperation aimed at collecting and analyzing genomic and health data from 500,000 participants in Finnish biobank. FinnGen Study is one of the earliest large individualized medical projects of this scale, which combines genomic information with digital health care data, aiming to improve human health through genetic research, provide novel medical diagnosis and treatment insights, and build world-class resources for future research. The GWAS data of EHT from FinnGen Study (18) Round 8 included 81,138 patients with EHT and 243,756 control cases of European ancestry. Detailed information about the data can be found here (https://risteys.finngen.fi/endpoints/I9_HYPTENSESS).

UK Biobank (UKB) is a prospective cohort study of more than 500,000 volunteers aged between 40 and 69 recruited from the general population in the United Kingdom from 2006 to 2010. It is a large-scale GWAS data set worldwide, and researchers can screen clinical biomarkers through the genotype data. The GWAS data of EHT from UKB included a total of 463,010 adults, including 54,358 patients with EHT and 408,652 control cases. The above data is publicly available at the IEU OpenGWAS project (mrcieu.ac.uk).

The EHT GWAS data from UKB can be used to validate the MR analysis results of blood metabolites on EHT (from FinnGen) to enhance the reliability of the conclusion.

### 2.3 Selection of instrumental variables

This study carried out many steps to select IVs strongly related to blood metabolites. Based on the correlation hypothesis, IVs were extracted from 486 blood metabolites using *P *< 1 × 10^−5^ as the significance threshold, consistent with many previous studies (19–21). Then, the European genotype of 1000 Genomes was used as a reference panel to perform linkage disequilibrium (LD) analysis, with LD parameters set to *r*^2^ of 0.1 and the distance of within 500 kb. Then, the SNPs that were associated with exposure were extracted from the outcome data of EHT, whereas the SNPs that were associated with the outcome were excluded (*P* < 1 × 10^−5^). And then, exposure SNPs and outcome SNPs were harmonized, and the SNPs containing incompatible alleles and palindrome SNPs with medium effective allele frequency were excluded. To determine if there was a weak IVs bias, we calculated the F-statistic of each metabolite to quantitatively assess the strength of IVs, where values <10 were indicative of weak IVs and would be eliminated to ensure that all SNPs have sufficient statistical strength for the corresponding metabolites (22).

### 2.4 MR analysis

The major MR analysis tool used to determine causality in this study was the inverse-variance weighted (IVW) model (23). IVW is the most widely used method in MR study, which assumes that all genetic variations are valid IVs. When IVs satisfy three hypotheses and are not affected by pleiotropy, it can provide an efficient and consistent estimate of the causal effect of the exposure on the outcome. Results were considered statistically significant when *P *< 0.05.

### 2.5 Sensitivity analysis

Multiple sensitivity methods were used in this study. MR Egger (24), Weighted Median (25) and MR-PRESSO (26) were used as supplementary approaches for MR analysis. These approaches employ diverse hypothesis models to analyze causation, and the consistency in the directions of various MR analysis models enhances the confidence of causal inference. Heterogeneity was assessed by Cochran's Q test, and *P *< 0.05 was used to indicate the presence of heterogeneity (27). MR Egger intercept (28) and MR-PRESSO global test were used to detect the potential horizontal pleiotropy. When the statistical threshold is less than 0.05, the MR analysis will be affected by horizontal pleiotropy. MR-PRESSO global test can also identify the outliers of pleiotropy. After that, Leave-one-out analysis was performed, the fundamental idea behind which is to perform the elimination of each SNP in a sequential manner and then compute the meta-effect of the SNPs that were left. If the results are significantly changed after excluding a certain SNP, it suggests that the significant results may be driven by individual SNP (29).

In addition, we calculated the power of MR using the mRnd method (https://hiny.cnsgenomics.com//mRnd/) (30).

All analyses were performed using R (version 4.2.3) and “TwosampleMR”, “Mendelianmization” and “MR-PRESSO” R packages.

### 2.6 Replication analysis

Repeated analysis of additional EHT GWAS data (from UKB) further confirmed the robustness of the candidate blood metabolites in this investigation. Then the results of the two analyses were comprehensively compared.

### 2.7 Direction validation

In this study, the Steiger test (31) was used to confirm whether the causal relationship between blood metabolites and EHT was biased because of reverse causality. When the phenotypic variation in EHT explained by the IVs was lower than that of the blood metabolites (Steiger *P* < 0.05), it indicated that the assumed direction of causality was correct.

### 2.8 Metabolic pathway analysis

MetaboAnalyst 5.0 (https://www.metaboanalyst.ca/) was used to examine the screened blood metabolites and investigate the relationship between metabolic pathways and EHT. The dataset for pathway analysis was obtained from the Small Molecular Pathway Database (SMPDB) and KEGG.

The following requirements were necessary for this investigation to establish a robust causal link between blood metabolites and EHT: First, *P* < 0.05 was set as the significance threshold for IVW analysis; Second, there was no evidence of horizontal pleiotropy or heterogeneity by sensitivity analysis; Third, the results were verified by replication analysis; Fourth, the causal direction was verified.

## 3 Results

### 3.1 Effects of genetically determined metabolites on essential hypertension

Among 486 blood metabolites, each blood metabolite had 3–244 SNPs. All F-statistics were greater than 10, proving our IVs were effective for MR analysis.

IVW method was used to evaluate the causal relationship between 486 blood metabolites and EHT (from FinnGen). Results showed that 45 metabolites related to EHT were detected (*P *< 0.05), including 30 known and 15 unknown metabolites. Among them, known metabolites included 12 amino acids, ten lipids, two sugars, two peptides, one cofactor and vitamin, one energy product and two xenobiotics. (see Supplementary Table S1).

**Figure 2:**
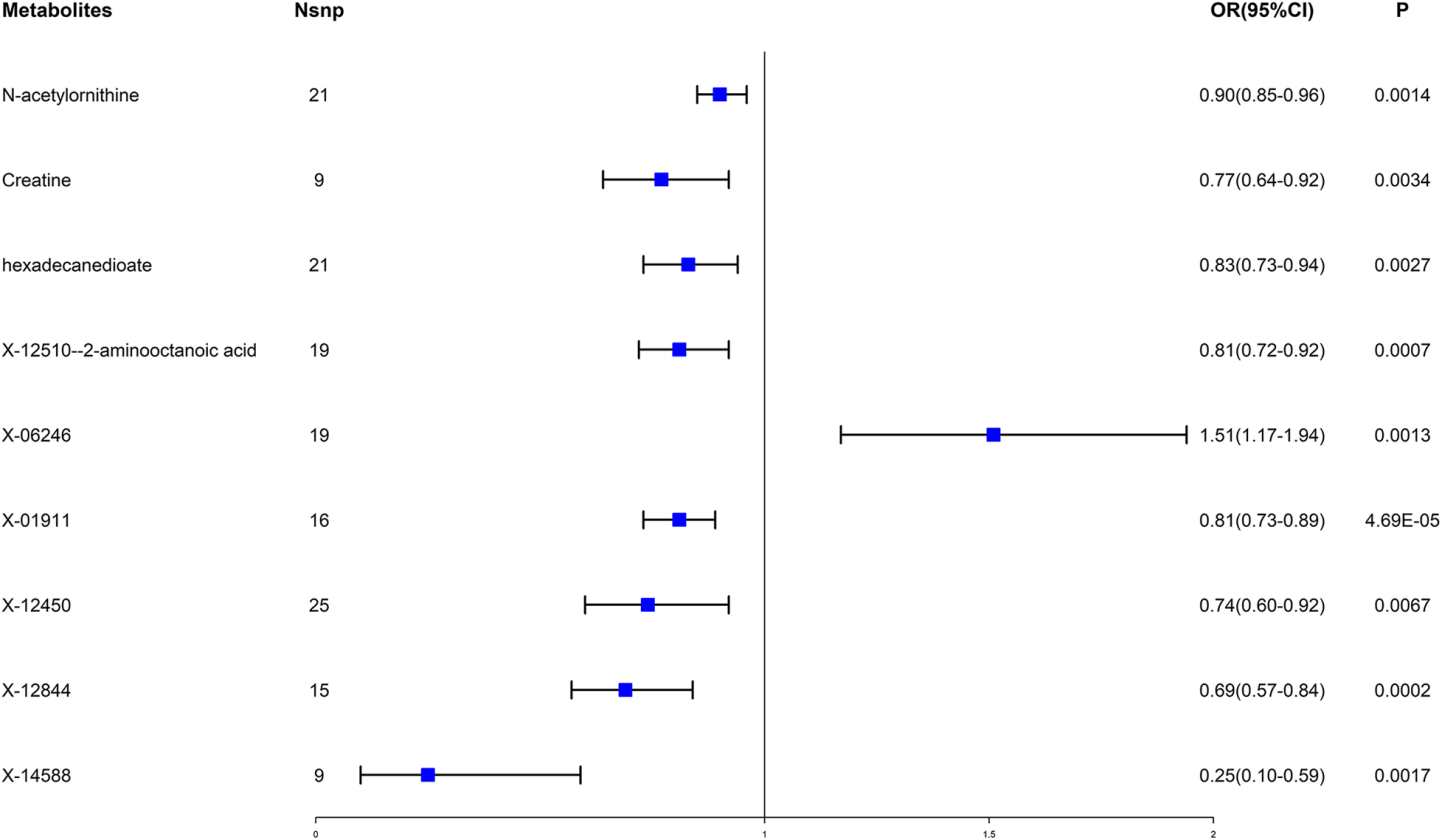
Forest plot for the causal effect of blood metabolites on essential hypertension. OR, odds ratio; CI, confidence interval.

Then many different sensitivity analyses were carried out to evaluate the reliability of the results (see Supplementary Table S2; Supplementary Figure S1). Under the stringent screening circumstances, nine blood metabolites (including three amino acids, one lipid, and five unknown metabolites) were ultimately selected. Among these metabolites, one unknown metabolite X-19364 was associated with the increased risk of EHT; The other eight metabolites were shown to be related to the reduced risk of EHT, including N-acetylornithine (odds ratio (OR) = 0.90, 95% confidence interval (CI) = 0.85–0.96, *P* = 0.0014), creatine (OR = 0.77, 95% CI = 0.64–0.92, *P* = 0.0034), hexadecanedioate (OR = 0.83, 95% CI = 0.73–0.94, *P* = 0.0027), X-12510–2-aminooctanoic acid (OR = 0.81, 95% CI = 0.72–0.92, *P* = 0.0007) and four unknown metabolites. (see Figure 2).

Finally, the mRnd method was used to estimate the statistical power of the above nine metabolites on EHT. The results were all 100%, thus confirming the strong causal relationship between candidate blood metabolites and EHT.

### 3.2 Replication analysis and results validation

**Figure 3:**
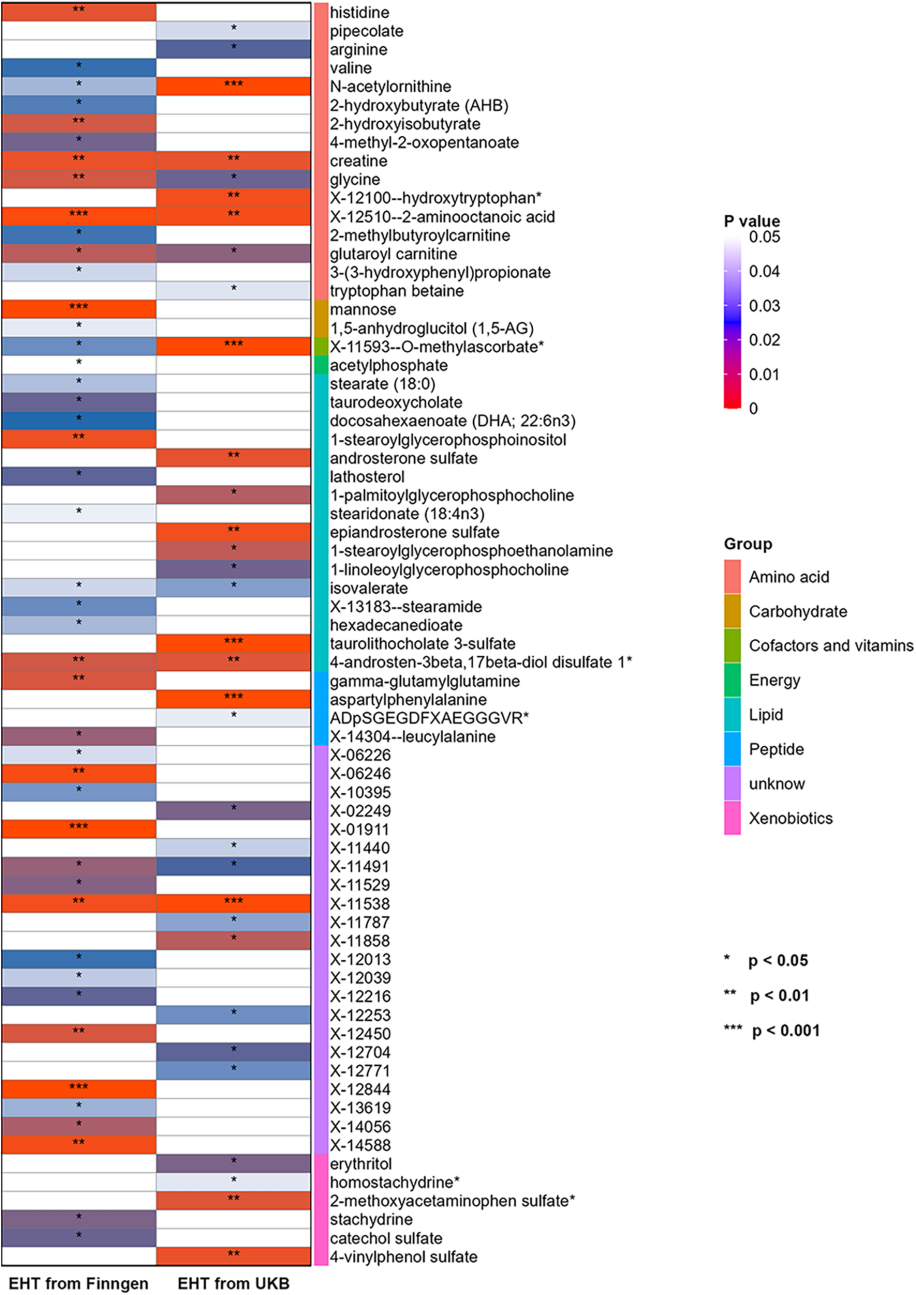
Heatmap for the causal estimate of blood metabolites (derived from the IVW analysis) on EHT. EHT, essential hypertension; IVW, inverse-variance weighted; UKB, UK Biobank.

Another set of GWAS data of EHT was obtained from UKB and subjected to repeated analysis using the same MR research approach in order to further confirm the findings. Through IVW analysis, 33 blood metabolites were screened (see Figure 3).

Then sensitivity analysis was conducted, and nine blood metabolites related to EHT were finally screened, including two lipids, three amino acids, one peptide, one xenobiotic, and two unknown metabolites. (see Supplementary Table S3).

**Figure 4:**
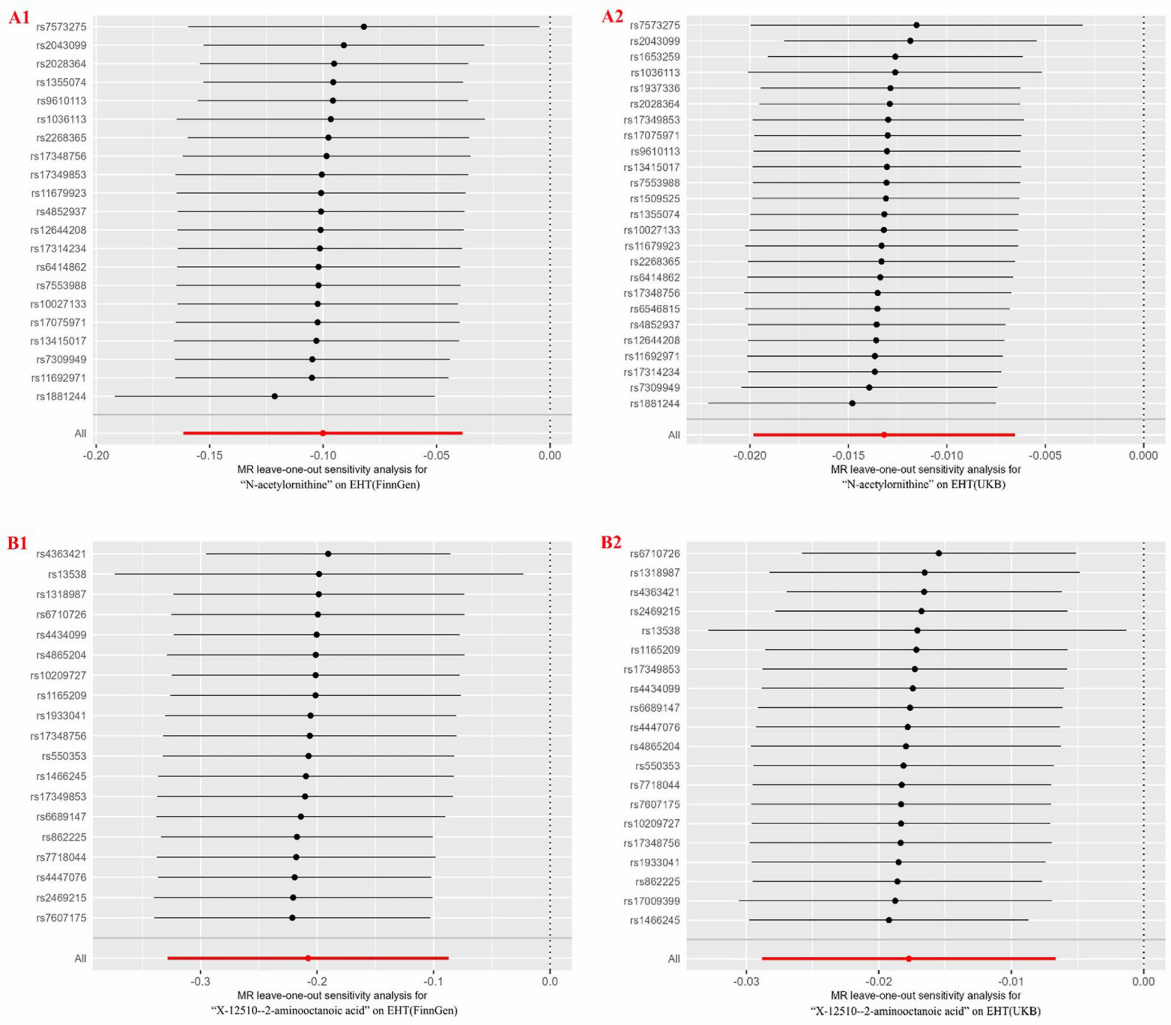
Forest plots for leave-one-out analysis of overlapped metabolites for two different GWAS datasets of EHT. (**A1**, **A2**) N-acetylornithine on EHT; (**B1**, **B2**) X-12510-2-aminooctanoic acid on EHT. EHT, essential hypertension; GWAS, genome-wide association study; UKB, UK Biobank.

Two blood metabolites, N-acetylornithine and X-12510-2—aminooctanoic acid, were discovered to overlap when compared with previous MR analysis results (EHT GWAS data from FinnGen). Among them, N-acetylornithine was negatively correlated with the risk of EHT (OR = 0.987, 95% CI = 0.980–0.993, *P *= 1.01 × 10^−4^). The Q value and *P* value of Cochran's Q test were 31.7586 and 0.1331; The RSSobs value and *P* value of the MR-PRESSO global test were 33.799 and 0.1788; The intercept value and *P* value of the MR-egger intercept test were 3.72 × 10^−5^ and 0.9045. It was indicated that there was no heterogeneity and pleiotropy. According to the results of the Leave-one-out analysis, a single SNP did not have a significant impact on the causality evaluation (see Figure 4). It was suggested that N-acetylornithine had a robust causal relationship with the reduction of risk of EHT. X-12510-2-aminooctanoic acid is an insufficiently defined aminooctanoic acid metabolite. The analysis results suggested that it had a protective effect on EHT (OR = 0.982, 95% CI = 0.972–0.993, *P *= 0.0017). The sensitivity analysis results showed that the Q value and *P* value of Cochran's Q test were 27.9027 and 0.0853; The RSSobs value and *P* value of MR-PRESSO global test were 29.3388 and 0.1434; The intercept value and *P* value of the MR trigger intercept test were −5.6 × 10^−5^ and 0.8880. leave-one-out analysis showed that the significant result was not driven by a single SNP (see Figure 4).

### 3.3 Direction validation

Steiger test was conducted to confirm the causal direction of candidate blood metabolites on EHT, and the results were all true. And Steiger *P* values were all less than 0.001, indicating that the determined causality was not biased due to reverse causality. (see Table 2).

**Table 2:** Steiger direction test of overlapped metabolites on essential hypertension.

Exposure	N-acetylornithine	X-12510-2-aminooctanoic acid
Correct causal direction	True	True
Steiger *P* value	<0.001	2.33 × 10^−306^

### 3.4 Metabolic pathway analysis

Five metabolic pathways connected to EHT were found by metabolite pathway analysis of blood metabolites obtained from IVW (*P* < 0.05), including “Valine, leucine and isoleucine biosynthesis” (*P *= 0.001), “Aminoacyl-tRNA biosynthesis” (*P *= 0.004), “Glycine, serine and threonine metabolism” (*P *= 0.021), “Biosynthesis of unsaturated fatty acids” (*P *= 0.025) and “Valine, leucine and isoleucine degradation” (*P *= 0.031).

## 4 Discussion

We first identified nine blood metabolites (five unknown and four recognized) among 486 blood metabolites that may have causal associations with EHT. Then, in order to confirm the findings, we ran a replication analysis using data from an additional EHT GWAS source. Lastly, we found two overlapped metabolites (N-acetylornithine and X-12510-2–aminooctanoic acid), both of which had causal relationships with the reduction of the risk of EHT.

Hypertension can cause multiple target organ damage, complicated by coronary artery disease, diabetes, stroke and other diseases, which is the most important risk factor for cardiovascular and cerebrovascular diseases. At present, the incidence of hypertension has been increasing worldwide, which has caused a heavy burden on human society. Although there are many drugs available to treat hypertension, many patients who receive treatment fail to achieve target blood pressure, which can be partially attributed to the complexity of different blood pressure regulation mechanisms in each individual. Although there have been many studies on the pathogenesis and pathological mechanisms of EHT, including endocrine system disorders (32), vascular endothelial cell dysfunction (33, 34), nervous system dysfunction (35), oxidative stress (36), insulin resistance (37), etc., the specific mechanism remains unclear. Therefore, it is very meaningful and necessary to elucidate the pathophysiology of hypertension, uncovering novel biomarkers and determine new biological pathways.

In this study, the two overlapped blood metabolites N-acetylornithine and X-12510-2–aminooctanoic acid all belong to amino acid derivatives, indicating that amino acid metabolism is the key part of EHT, which is consistent with a previous study (38). According to a prior research, abnormal metabolism of amino acids has been linked to cardiovascular disorders (39). And an early study suggested that EHT may be an inherited disorder in amino acid metabolism (40).

The results of this study suggested that N-acetylornithine had a protective effect on EHT, but there were limited studies on the correlation between N-acetylornithine and hypertension. N-acetylornithine, with the molecular formula C_7_H_14_N_2_O_3_, is an n-acylated α-amino acid with L-configuration of α-carbon atoms and a tautomer of a N(2)-acetyl-L-ornithine zwitterion, belonging to a class of organic compounds called n-acyl-l-α-amino acids, which are found in all living things, including bacteria, plants, humans, etc. N-acetylornithine is an important intermediate of arginine metabolism (41). Arginine is a basic amino acid that helps make protein and is also the building block for creatine, putrescine, and nitric oxide. Many studies have found a close relationship between arginine and hypertension. In patients with hypertension, the levels of arginine were low (42), and arginine supplementation could lower blood pressure levels (43–46). Arginine synthesizes nitric oxide by nitric oxide synthase, and nitric oxide plays a vital role as an intercellular messenger and neurotransmitter in the cardiovascular system and nervous system, including promoting vasodilation, inhibiting platelet aggregation, inhibiting smooth muscle cell proliferation and so on (47). When the arginine level decreases, the content of nitric oxide in the blood vessel wall decreases, which makes the blood vessels lack enough relaxation, leading to the narrowing of the arterials, thus causing the increase of blood pressure. Besides, N-acetylornithine is an important biomolecule in the synthesis of ornithine. Ornithine is closely related to arginine. The vascular tissue expresses arginase, which metabolizes arginine into ornithine and urea, thus reducing the substrate utilization rate of nitric oxide formation (48, 49). Ornithine, as an arginase inhibitor, had been reported to reduce elevations in diastolic and systolic blood pressure (50). And ornithine was found significant to identify patients at heart failure stage C (51). Therefore, N-acetylornithine may have a protective effect on hypertension through arginine or ornithine pathway.

Metabolomics is a method to study biochemical processes through a comprehensive analysis of metabolites in organisms, which provides important tools and insights for understanding the metabolic mechanism of diseases, identifying new treatment targets and monitoring treatment results (52). In this study, it is found that N-acetylornithine has a protective effect on EHT, which has potential guiding significance for the pathogenesis and therapeutic targets of hypertension. N-acetylornithine is hydrolyzed to ornithine and acetic acid by specific enzymes such as N-acetylornithine, and ornithine is converted to arginine in the urea cycle (53). Arginine is catalyzed by nitric oxide synthase to produce nitric oxide and citric acid (54). The above pathways show the complex biochemical processes from amino acid derivatives to the important biological signal molecule nitric oxide. Nitric oxide, the final product of this pathway, is an important vasodilator, which is produced in vascular endothelial cells to help relax vascular smooth muscle, thereby reducing blood pressure, and has become an important target for the treatment of cardiovascular diseases. For example, drugs such as nitroglycerin and nitroprusside cause vasodilation by releasing nitric oxide in the body, thereby exerting an antihypertensive effect (55).

X-12510-2-aminoooctanoic acid belongs to a kind of 2-aminooctanoicacid, but its composition is not fully defined. Aminooctanoicic acid is the main component of protein and is an essential amino acid in living organisms. 2-Aminooctanoic acid is an aliphatic amino acid with an amino acid group and is not an essential amino acid for the human body. It is a secondary metabolite that may function as a defense or signal molecule. In certain circumstances, these metabolites are not significant in metabolism or physiology. Therefore, the correlation we found between it and hypertension in our study may be coincidental. Additionally, its specific components need to be further clarified.

Creatine and hexadecanedioate were statistically significant in the MR analysis of blood metabolites on EHT (from FinnGen). However, these findings were not conclusively confirmed in the replication analysis. The possible reason for this discrepancy is that the hypertensive populations from the UKB and FinnGen are from different countries, and the number of individuals in each dataset is also inconsistent. Additionally, the disease states may vary between these populations, leading to some differences in the results. Nonetheless, we can still analyze the relationship between creatine, hexadecanedioate, and hypertension. The main metabolic function of creatin is to combine creatine kinase with phosphate groups to produce phosphocreatine, which is used to regenerate adenosine-triphosphate (ATP) to meet the energy demand. Cellular energy transport relies on the creatine-phosphocreatine pathway, and a deficit in creatine is indicative of an issue with energy metabolism (56). In our study, the level of creatine was negatively correlated with EHT, indicating that myocardial energy was insufficient to produce enough ATP to meet the body's energy demands. Ioanna et al. found that creatine concentration was inversely proportional to blood pressure (57). Our results showed that creatine was negatively associated with EHT, which was consistent with the above study. Hexadecanedioate, also known as a,omega-hexadecanedioic acid or c16DCA (2-), is a kind of compound called long-chain fatty acid, which is the secondary pathway of fatty acid oxidation when β-oxidation is insufficient. Hexadecanedioate was linked to elevated blood pressure by Menni et al. (58–60), but our findings revealed that it was linked to a lower risk of hypertension. The possible reason for this may be that we studied hypertension, while the Menni team studied blood pressure values, so there may be some inconsistencies. A more convincing RCT may be needed for further investigate and verification.

Previously, Qiao et al. (61) and Dai et al. (62) conducted MR analyses on blood metabolites and blood pressure, focusing on systolic blood pressure (SBP) and diastolic blood pressure (DBP). However, this study targets EHT, indicating a difference in study subjects. Analyzing hypertension as a whole or examining SBP and DBP separately is highly valuable, as these approaches address key questions from different perspectives. Integrating studies on the relationship between these metabolites and hypertension can significantly contribute to the advancement of this field. Additionally, this study adopted a more rigorous approach compared to the previous two studies. Beyond the conventional MR analysis, it introduced another independent hypertension dataset for replication analysis and results validation, enhancing the credibility of the identified metabolites. Furthermore, multiple sensitivity analysis methods were employed, the power of MR was calculated, and direction was validated to highlight the robustness of the analysis results from multiple perspectives.

In the MR analysis by Qiao et al. (61), three metabolites, namely N-acetylglycine (OR = 0.946, 95% CI: 0.920–0.973, FDR = 0.023), X-09026 (OR = 0.845, 95% CI: 0.779–0.916, FDR = 0.021), and X-14473 (OR = 0.938, 95% CI: 0.907–0.971, FDR = 0.040), were found to be associated with DBP. However, no metabolites significantly correlated with SBP. In the MR analysis by Dai et al. (62), 12 metabolites were significantly associated with DBP and 22 metabolites with SBP. Sensitivity analysis revealed that four metabolites, namely 1-(1-enyl-stearoyl)-2-arachidonoyl-GPE (P-18:0/20:4)* (OR = 0.79, 95% CI: 0.70–0.88, *P* = 3.78^−05^), behenoyl dihydrosphingomyelin [d18:0/22:0]* (OR = 1.84, 95% CI: 1.39–2.44, *P* = 2.04^−05^), N-acetylarginine (OR = 1.15, 95% CI: 1.08–1.22, *P* = 1.17^−05^), and N-acetylglutamine (OR = 1.15, 95% CI: 1.08–1.22, *P* = 1.17E^−05^), were significantly associated with DBP, and one metabolite, namely X-11381 (OR = 1.58, 95% CI: 1.28–1.94, P = 1.58^−05^), was examined for its significant association with SBP. The results of these two studies differ, possibly due to differences in the metabolite datasets and hypertension datasets they selected. In our study, N-acetylornithine and X-12510-2–aminooctanoic acid were found to be closely related to EHT. Considering these three analyses together, they all involve derivatives of amino acids. N-acetylglycine, N-acetylarginine, N-acetylglutamine, and N-acetylornithine are four different N-acetylated amino acid derivatives, formed by adding an acetyl group (-COCH3) to the amino (-NH2) group of the respective amino acids. N-acetylation is a common post-translational modification of proteins that affects protein stability, activity regulation, and interactions with other cellular components. These N-acetylated amino acid derivatives are involved in various biochemical pathways, and due to their structural differences, they have different specific functions in organisms. Although these molecules have different functional emphases, they generally play roles in regulating intracellular environments, metabolic balance, and signal transduction, which may affect the development of hypertension through the biological functions they exert. N-acetylglycine has not been definitively identified as a direct regulator of hypertension, but as part of amino acid metabolism, it may indirectly participate in blood pressure regulation by affecting metabolic states, antioxidation, and neural regulation. N-acetylarginine is an N-acetylated derivative of arginine, which plays an important role in the generation of nitric oxide, a key vasodilator that directly participates in blood pressure regulation. N-acetylglutamine, as a derivative of glutamine, may indirectly influence blood pressure through metabolic health, antioxidative effects, and immune regulation. Our future research can focus on these amino acid metabolites and conduct experimental studies for further analysis and validation.

This study had some strengths. First, MR analysis of 486 blood metabolites on EHT is a very comprehensive and systematic study. Second, MR research significantly reduced traditional studies’ observation bias and had no reverse causality or confounding effects. Third, the replication analysis using another source of EHT GWAS data to verify the results could more strongly explain the causal effect of candidate metabolites on EHT.

This study also had some limitations. First, in order to avoid biases caused by genetic heterogeneity, all the population of GWAS in this study were from Europe, so it is uncertain whether the same results would have been produced for different ethnic groups. Second, the range of the P threshold was relaxed due to the limited number of SNPs reaching genome-wide significance for blood metabolites, but F values for all SNPs were above 10 (to exclude weak instrumental variables), which is a widely used research method. Third, some of the blood metabolites obtained from IVW analysis are unknown metabolites, whose structure and function are still unclear, so detailed analysis cannot be conducted.

## 5 Conclusion

In conclusion, this study conducted the two-sample MR method to examine the causative association between 486 blood metabolites and EHT and screened out metabolites having predictive value for EHT, including N-acetylornithine, which caused EHT. This study improves our understanding of blood metabolites and EHT and seeks to shed light on hypertension’s pathophysiology.

## Data Availability

The original contributions presented in the study are included in the article/**Supplementary Material**, further inquiries can be directed to the corresponding author.
