# Supplementary material for: Causal association between blood metabolites and risk of hypertension: a Mendelian randomization study
Source: Front Cardiovasc Med. 2024 Jun 7;11:1373480. doi: 10.3389/fcvm.2024.1373480 (PMC11190327; doi:10.3389/fcvm.2024.1373480)

**Figure S1** Leave-one-out plots for blood metabolites on the risk of essential hypertension based on the inverse variance weighted (IVW) method


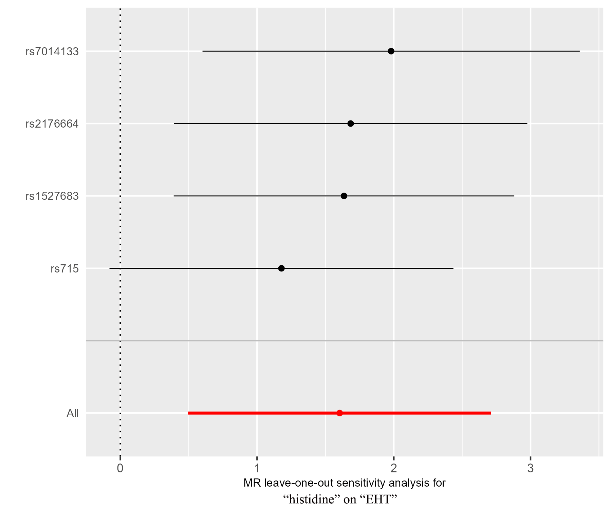

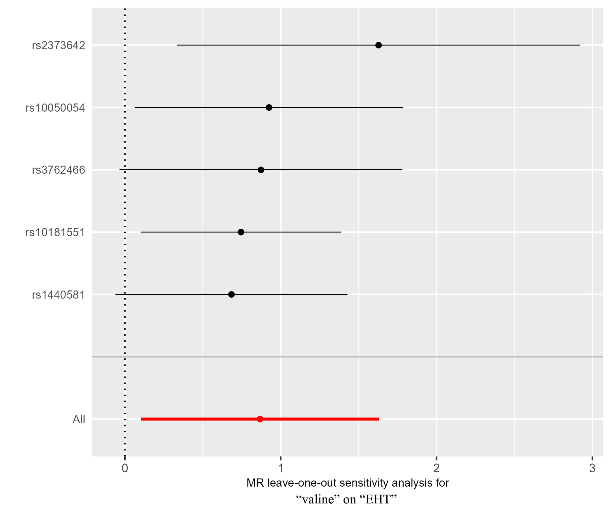

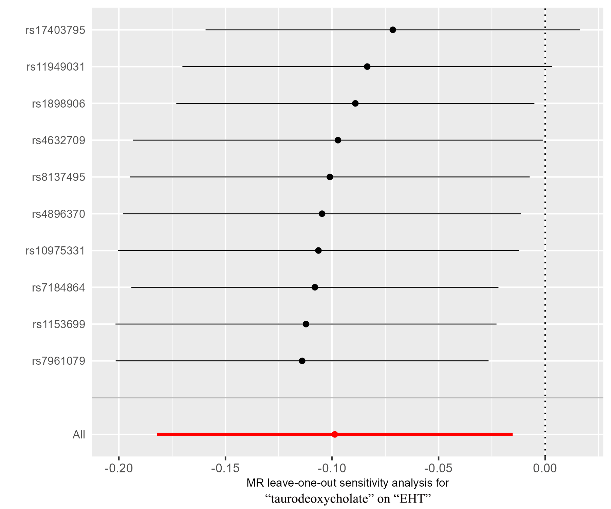

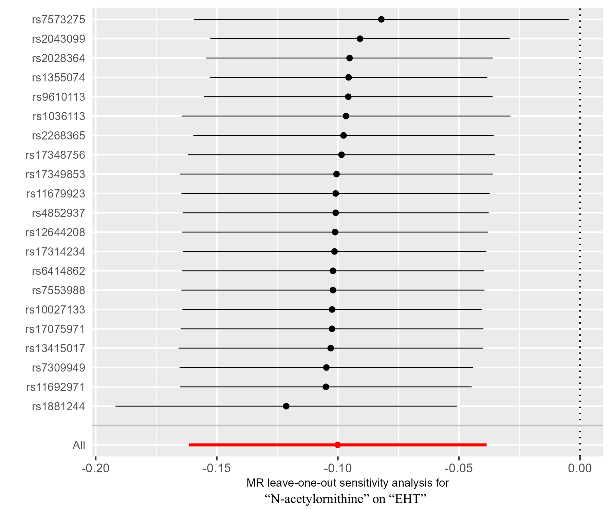

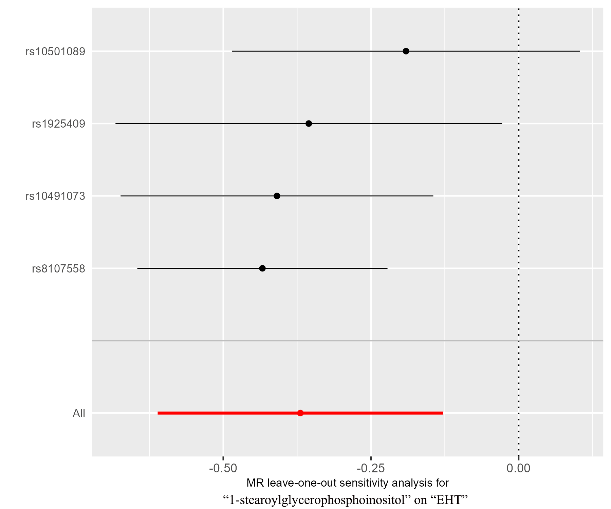

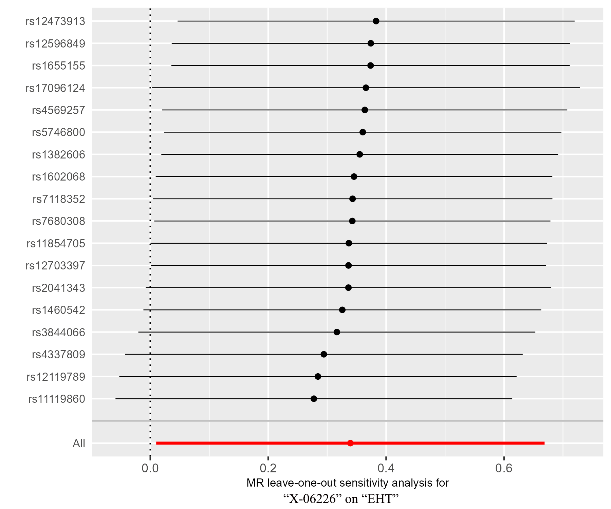

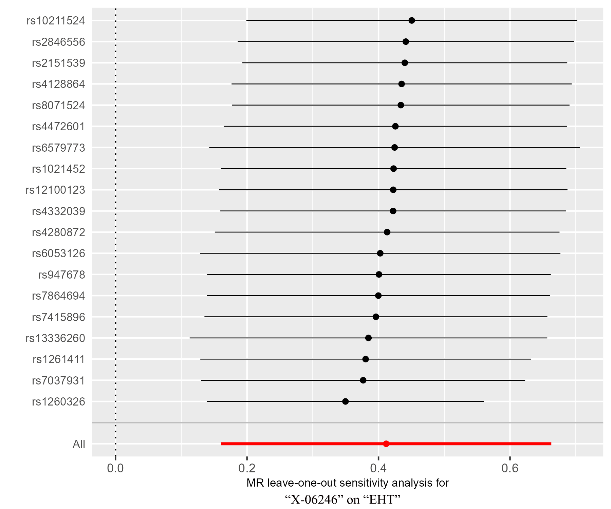

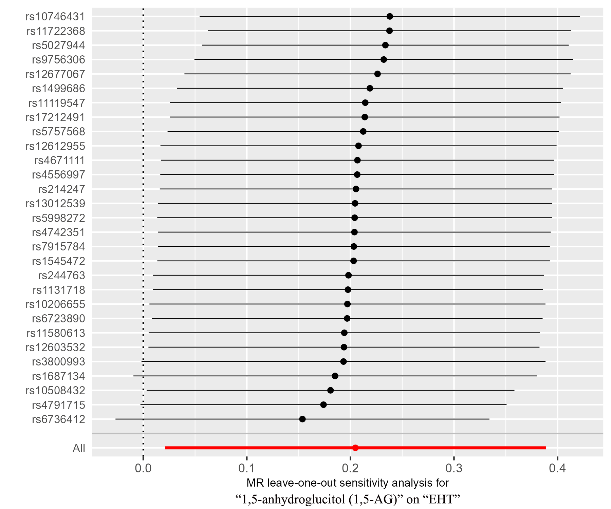

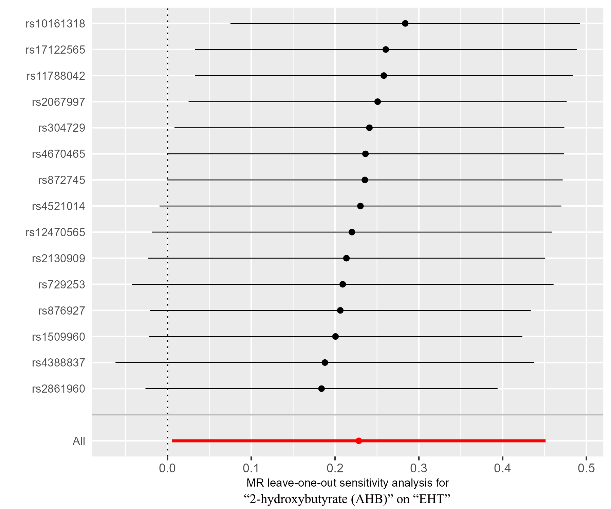

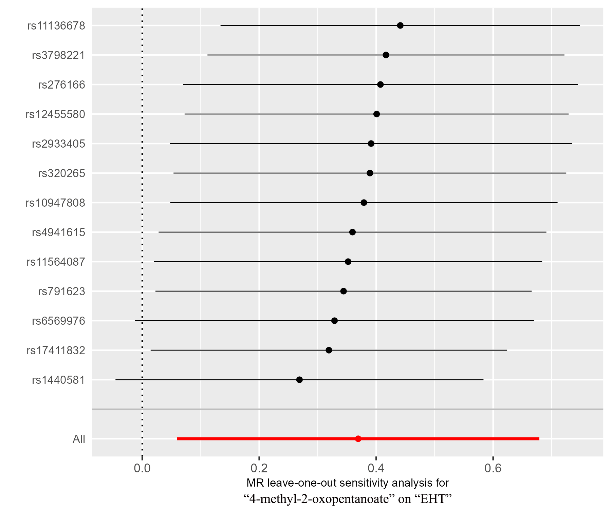

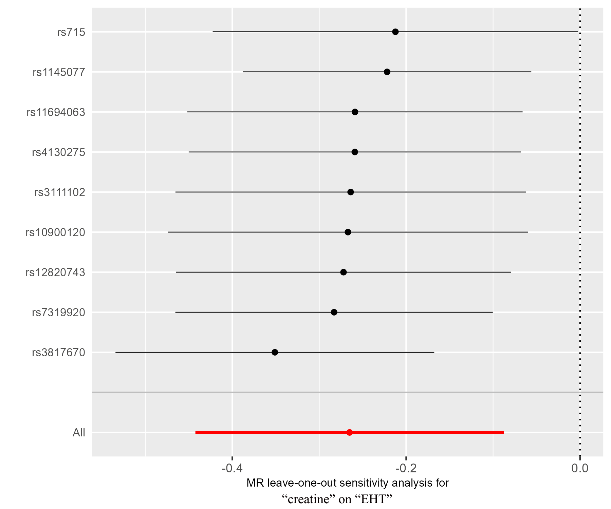

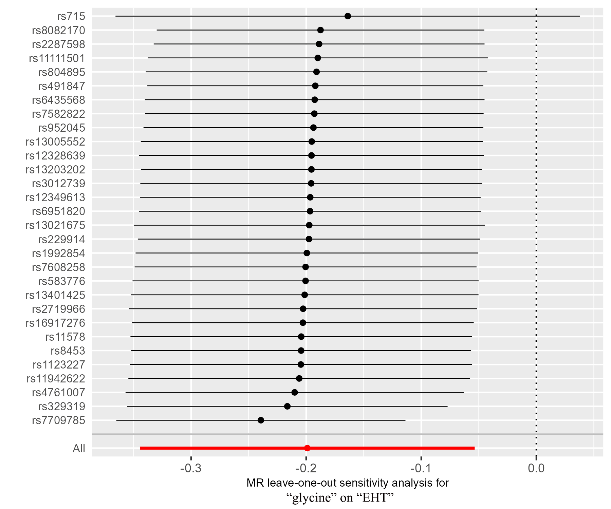

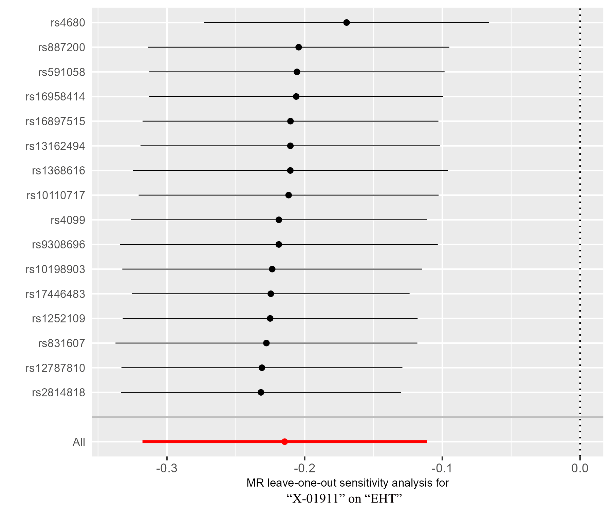

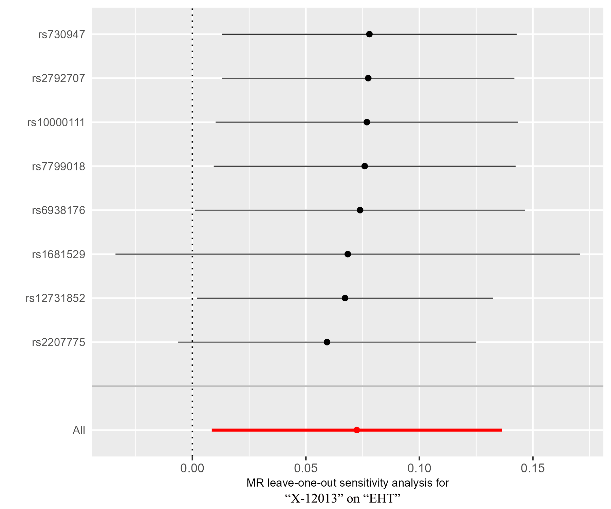

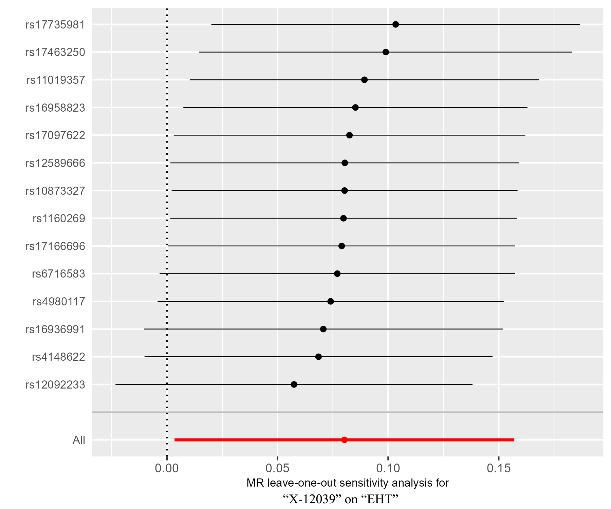

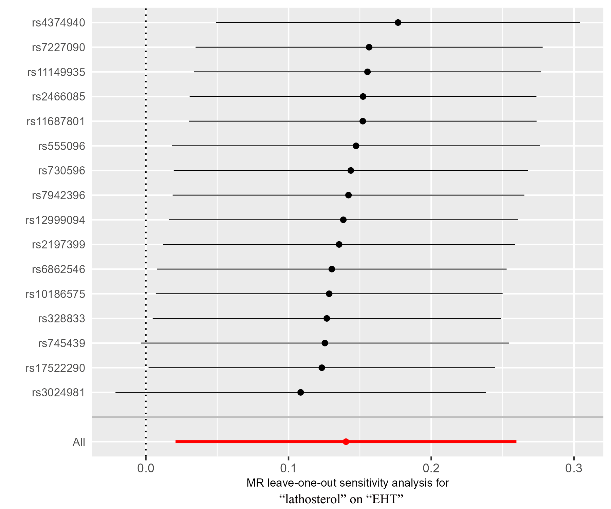

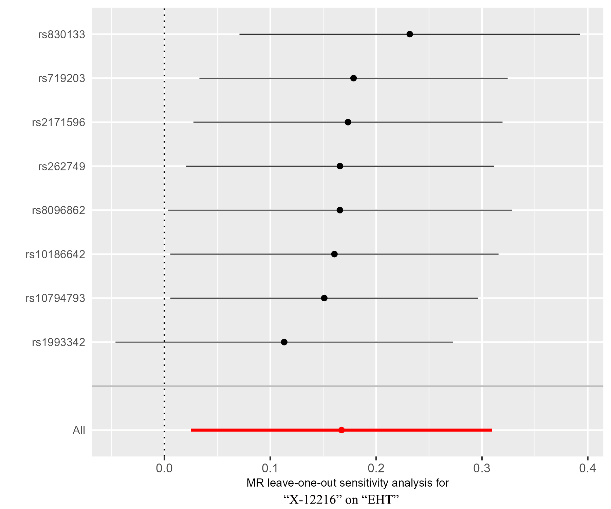

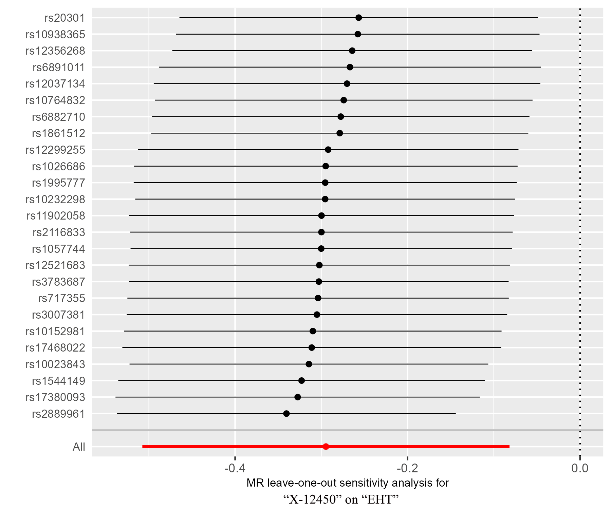

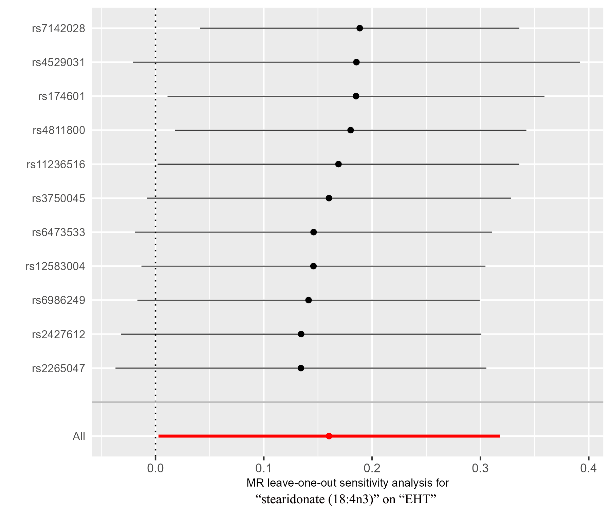

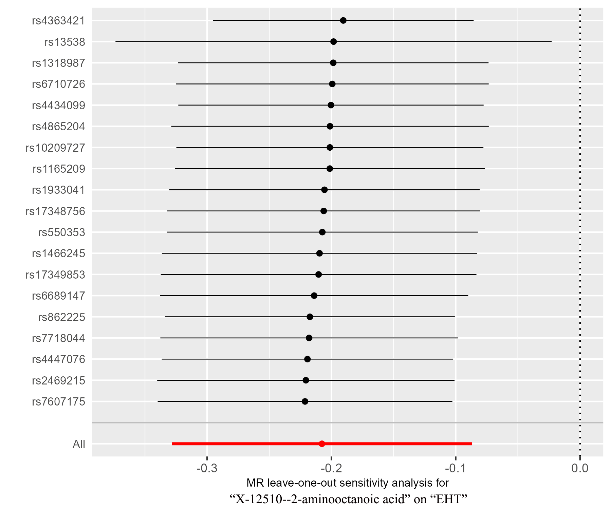

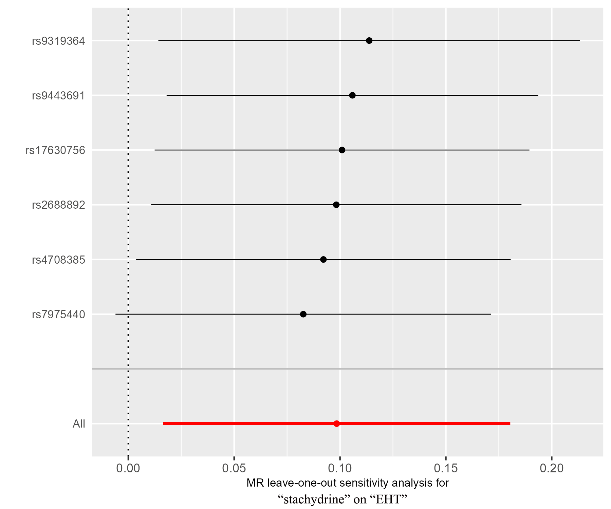

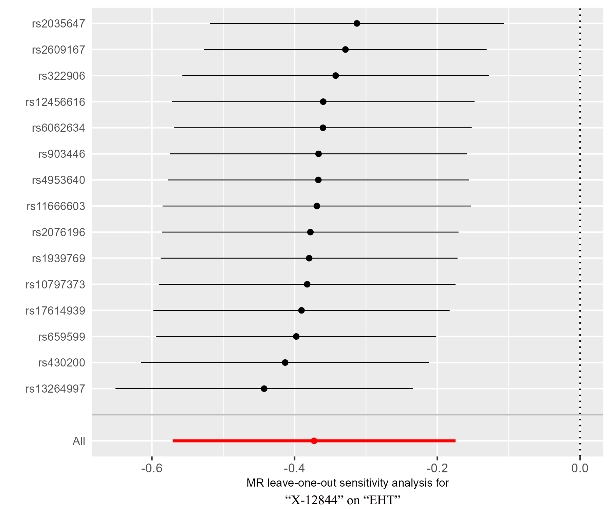

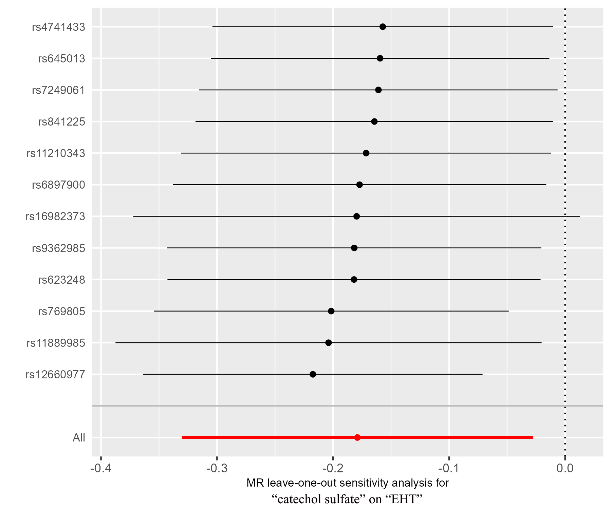

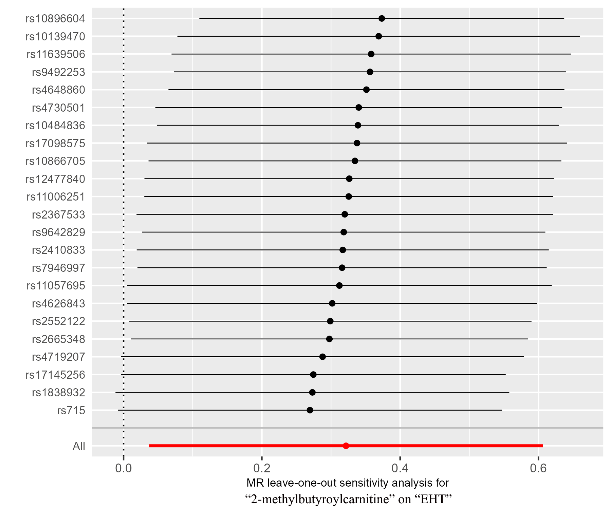

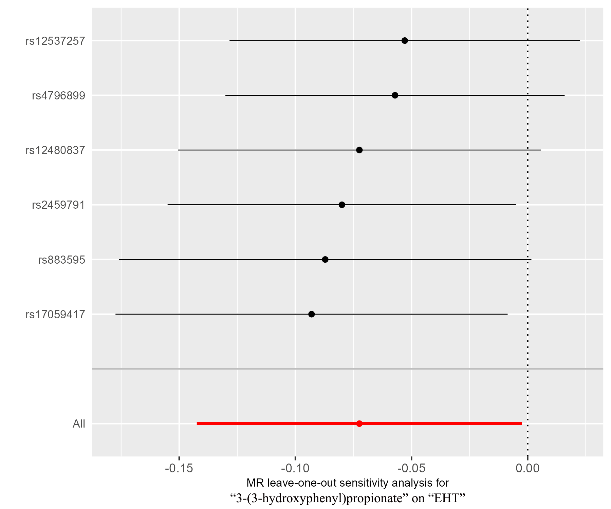

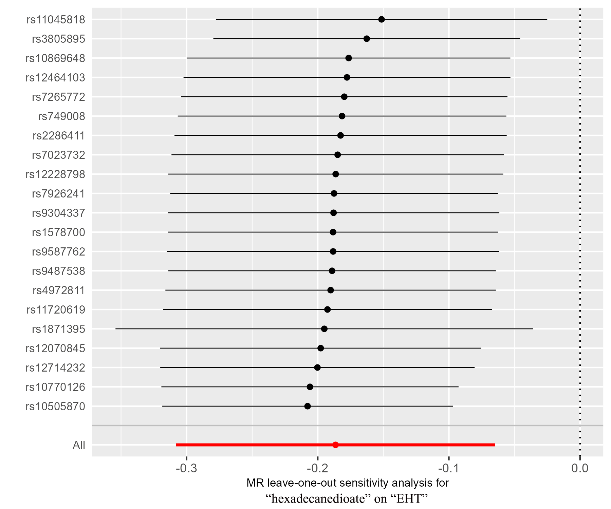

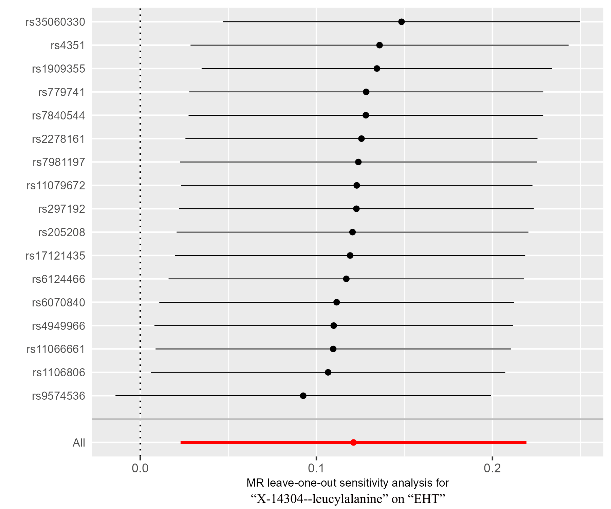

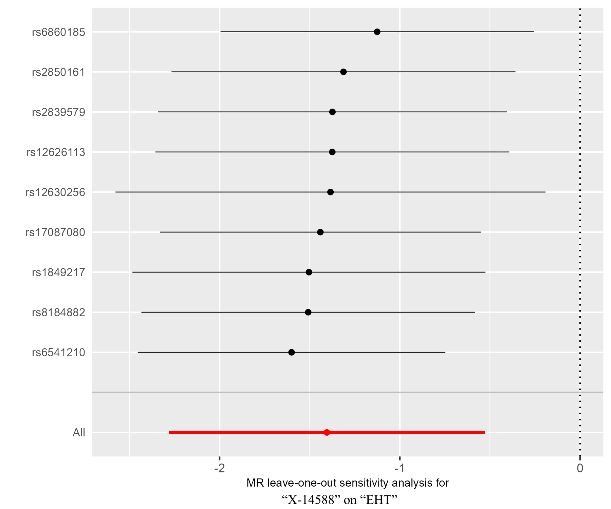

Supplement: Supplementary file 2 [file Datasheet1.docx]
